# Do Electrocardiogram Rhythm Findings Predict Cardiac Activity During a Cardiac Arrest? A Study from the Sonography in Cardiac Arrest and Hypotension in the Emergency Department (SHoC-ED) Investigators

**DOI:** 10.7759/cureus.3624

**Published:** 2018-11-23

**Authors:** Paul R Atkinson, Andrew W Keyes, Kathleen O'Donnell, Nicole Beckett, Ankona Banerjee, Jacqueline Fraser, David Lewis

**Affiliations:** 1 Emergency Medicine, Saint John Regional Hospital, Saint John, CAN; 2 Emergency Medicine, Saint John Regional Hospital / Horizon Health Network, Saint John, CAN; 3 Miscellaneous, Memorial University, Saint John, CAN; 4 Internal Medicine, Saint John Regional Hospital / Dalhousie University, Saint John, CAN; 5 Epidemiology and Public Health, Saint John Regional Hospital / Horizon Health Network, Saint John, CAN; 6 Emergency Medicine, Dalhousie University, Saint John, CAN

**Keywords:** electrocardiogram (ecg), point-of-care ultrasound, point-of-care ultrasound, cardiopulmonary resuscitation, pocus, shoced

## Abstract

Introduction

Electrocardiographic (ECG) rhythms are used during advanced cardiac life support (ACLS) to guide resuscitation management. Survival to hospital discharge has been reported to be better for patients with pulseless electrical activity (PEA) than asystole in out-of-hospital arrests. Despite this, treatment for these two (non-shockable) rhythms is combined in ACLS guidelines. This study examines if the recorded cardiac rhythm of asystole or PEA during ACLS accurately predicts mechanical cardiac activity as determined by point-of-care ultrasound (PoCUS).

Methods

A database review was completed for patients (> 19 years without a do not resuscitate (DNR) order) who presented to a tertiary emergency department in PEA or asystolic cardiac arrest between 2010 and 2014. Patients were separated into two groups: those with electrical cardiac activity (PEA) and those without (asystole). We compared ECG rhythm and PoCUS-documented cardiac activity results (both initial and any) for each case.

Results

A total of 186 patients met the study criteria. The 46 patients with PEA on ECG were more likely to have cardiac activity than the 140 patients with asystole (odds ratio 7.22 (95% confidence intervals 2.79-18.7) for activity on initial PoCUS; odds ratio 5.45 (2.49-12.0) for activity on any PoCUS during arrest).

ECG alone was poorly sensitive for initial cardiac activity (63.64%; 40.66% to 82.80%) and any cardiac activity (54.29%; 36.65% to 71.17%), with specificity marginally better at 80.49% (73.59% to 86.25%) for initial and 82.12% (75.06% to 87.87%) for any activity.

Conclusion

Our results suggest that ECG rhythm alone is not an accurate predictor of cardiac activity. This supports the use of PoCUS during cardiac arrest, in addition to ECG, to identify patients with ongoing mechanical cardiac activity and to help determine appropriate treatment for this group.

## Introduction

**Figure 1:**
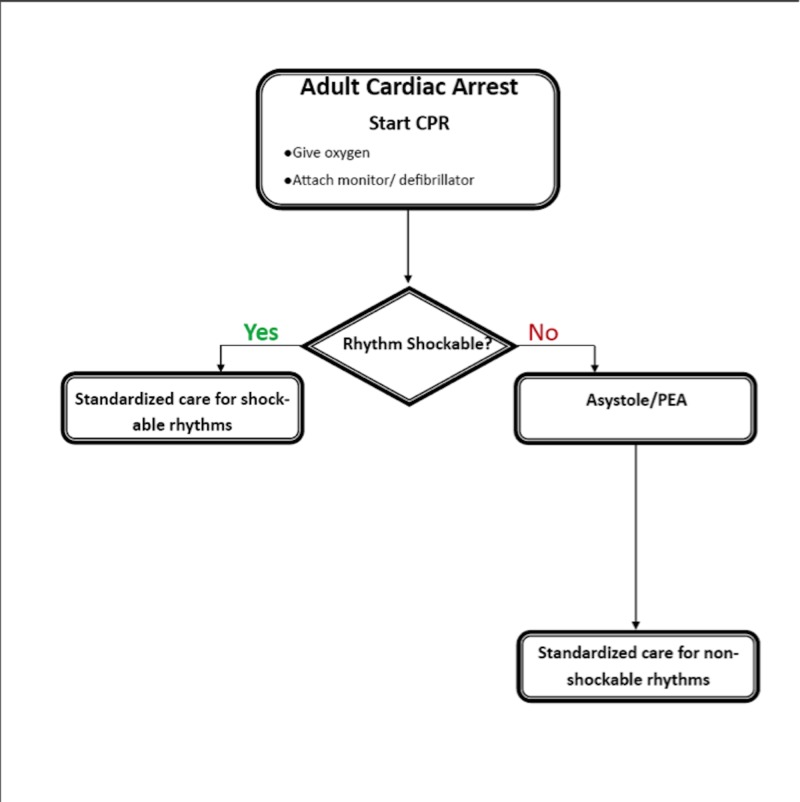
Typical resuscitation algorithm based on electrocardiogram rhythm CPR: Cardiopulmonary Resuscitation; PEA: Pulseless Electrical Activity; ECG: Electrocardiogram

Cardiac arrest is a significant cause of morbidity and mortality in North America; one out of every 7.4 people in the United States will die of sudden cardiac death [1]. Advanced Cardiac Life Support (ACLS) protocols, published by the American Heart Association (AHA), are the standard of care for cardiac resuscitation [1]. Medical professionals use the ACLS protocols to determine how or if they should proceed with resuscitation depending on patient presentation. Resuscitative action - whether that be defibrillation or medication administration, chest compressions, or termination of efforts - is frequently determined by findings on electrocardiogram (ECG) rhythm monitoring. ECG rhythms are continuously monitored during cardiac resuscitation, as they allow medical professionals to quickly and easily visualize cardiac activity (or the lack thereof) without disrupting resuscitative efforts. Despite our reliance on the ECG rhythm to determine the appropriate resuscitative pathway within ACLS algorithms (see Figure 1), it is unclear how well ECG findings correlate with ongoing mechanical cardiac activity in non-shockable cases, as there have been reports of cardiac activity, return of spontaneous circulation (ROSC), and even survival in patients that initially demonstrated asystole on ECG [2].

Within the last decade, point of care ultrasound (PoCUS) has become a key diagnostic tool for emergency physicians, as it allows them to quickly identify crucial diagnoses at the bedside [3]. PoCUS is now accepted as an adjunct during cardiac arrest. The 2015 update of the American Heart Association (AHA) Guidelines for Cardiopulmonary Resuscitation (CPR) and Emergency Cardiovascular Care [1] recommended that PoCUS be used during rhythm checks to assess myocardial contractility and to identify potentially treatable causes of cardiac arrests, such as severe hypovolemia, tension pneumothorax, pulmonary embolism, and cardiac tamponade, which historically have been diagnosed clinically. The International Federation for Emergency Medicine (IFEM) has published guidelines on the use of PoCUS during cardiac arrests [3].

The purpose of this study was to determine whether the presence of ECG electrical activity (PEA) accurately predicts the presence mechanical cardiac activity and whether the absence of ECG electrical activity (asystole) similarly predicts the absence of mechanical cardiac activity, as determined by PoCUS during the arrest. We reviewed cases of cardiac arrest in a tertiary hospital emergency department (ED) over a five-year period that employed both ECG and PoCUS to determine patients’ cardiac activity, using PoCUS as the reference standard to confirm cardiac activity.

## Materials and methods

Study settings

A database review was completed for patients who presented in cardiac arrest to the Saint John Regional Hospital (SJRH) emergency department (ED), a tertiary healthcare center in New Brunswick, Canada, between 2010 and 2014.

Subject selection

Patients were divided into two groups according to their initial emergency department ECG tracing: those with pulseless electrical activity (PEA) and those with asystole. Patients were further categorized based on PoCUS findings, i.e., whether or not cardiac activity was visualized on ultrasound. Patients who were younger than 19 years of age or had a previous do not resuscitate (DNR) order were excluded from the review.

Protocol for resuscitation

Resuscitation, delivered as routine care, was guided by ACLS protocols and institutional policies. PoCUS was performed during designated pauses, such as pulse and rhythm checks and necessary resuscitative procedures (e.g. intubation), so as to minimize cardiopulmonary resuscitation (CPR) interruption. Pauses were minimized as per ACLS recommendations, however, actual delays in CPR were not recorded.

Images were acquired using the standard PoCUS technique, using curvilinear or phased array ultrasound probes. Ultrasound views included subxiphoid, parasternal long axis, or apical four chambers. Image requirements were based on adequate echocardiographic windows and image quality, as determined by the physician performing the bedside ultrasound. For patients that were difficult to image, a combination of views was used to obtain adequate information. Sonographic images were obtained by competent personnel with experience in PoCUS; findings were communicated to the team leader.

Cardiac activity on PoCUS was defined as sustained coordinated contractility of the left ventricle, with visible valve movement.

Study outcomes

Our primary outcome was the diagnostic performance (diagnostic odds ratios, sensitivity, specificity, and likelihood ratios) for ECG rhythm findings as predictors of initial cardiac activity as determined by PoCUS. We also report any cardiac activity visualized on PoCUS performed during the arrest.

Data collection

The data for this study were obtained through a structured chart review. Data were taken from initial patient encounters, patient records, and emergency medical services (EMS) records (when available). Patients were included in the study if they met the selection criteria, i.e., adult patient (>19 yrs), without a DNR order, who suffered from a cardiac arrest and had cardiac activity determined by both ECG and PoCUS. Subject data, with Protected Health Information (PHI) removed, were stored in a local database. The local site kept secured records to enable the identification of the patient source if data review was required.

Patient information included the following: past medical history, events surrounding the cardiac arrest, actions taken by health care professionals, peri-arrest presentation, peri-arrest interventions, and patient outcomes. Recorded health care professional actions included ACLS medication administration, airway management, chest compressions, defibrillation, pacing, and other resuscitative interventions.

Statistical analysis

Data were analyzed by standard parametric and nonparametric tests using Prism for Mac (GraphPad Software, 2012, La Jolla, CA, US).

## Results

Of the charts reviewed, 186 patients met the study criteria; 140 with asystole and 46 with PEA recorded on the initial ECG (Table 1).

**Table 1:** Patient characteristics and outcomes PoCUS: Point of Care Ultrasound; ECG: Electrocardiogram; PEA: Pulseless Electrical Activity; SD: Standard Deviation; n: Number

	Cardiac Activity on Initial PoCUS	No Cardiac Activity on Initial PoCUS	Total	P-Value
Age (Mean; years +/- SD)	66.9 ± 2.77	64.34 ± 1.23	64.62 ± 1.14	0.7638
Sex (% Male)	40	69	60	0.0124*
Witnessed Arrest (n)	16	115	121	0.4392
PEA on ECG	14	32	46	<0.0001
Asystole on ECG	8	132	140	<0.0001
Total	22	164	186	

Patients with PEA on ECG were more likely to show mechanical cardiac activity on the initial PoCUS than those with asystole (odds ratio 7.22 (95% confidence intervals 2.78 to 18.7)) and for any PoCUS performed during the resuscitation (odds ratio 5.45 (24.48 to 12.0)). Of the 140 patients whose ECG showed asystole, eight (6%) had cardiac activity on PoCUS. Of the 46 patients with PEA, 14 (30%) had cardiac activity confirmed by PoCUS. ECG sensitivity alone was poorly sensitive for initial cardiac activity (63.64% (40.66% to 82.80%)) and for any cardiac activity (54.29% (36.65% to 71.17%)), with specificity marginally better (80.49% (73.59% to 86.25%) and 82.12% (75.06% to 87.87%)).

A summary of the specificity, sensitivity, positive and negative predictive value, likelihood ratios, and accuracy of ECG rhythm as a predictor for cardiac activity on initial and any PoCUS during cardiac arrest are reported in Table 2.

**Table 2:** Performance of electrocardiogram (ECG) rhythm as a predictor of initial cardiac activity (primary outcome) and any cardiac activity (secondary outcome) during cardiac arrest. PoCUS: Point of Care Ultrasound; CI: Confidence Intervals

Variable	Initial PoCUS Activity (95% CI)	Any PoCUS Activity (95% CI)
Sensitivity	63.64% (40.66% to 82.80%)	54.29% (36.65% to 71.17%)
Specificity	80.49% (73.59% to 86.25%)	82.12% (75.06% to 87.87%)
Positive Likelihood Ratio	3.26	3.04
Negative Likelihood Ratio	0.45	0.56
Positive Predictive Value	30.43% (21.93% to 40.53%)	41.30% (30.81% to 52.65%)
Negative Predictive Value	94.29% (90.43% to 96.65%)	88.57% (84.28% to 91.81%)
Odds Ratio	7.219 (2.789 to 18.677)	5.454 (2.488 to 12.0)

## Discussion

Our results demonstrate that ECG rhythm is not especially sensitive or specific as a predictor of mechanical cardiac activity during cardiac arrest. This is concerning, as ECG remains the gold standard for guiding resuscitation and determining cardiac activity during ACLS. With sensitivity just over 80% and an even lower specificity, it is clear that ECG rhythm cannot be relied upon to confirm functional asystole or ongoing cardiac activity. As such, our findings support the integration of PoCUS within ACLS algorithms to provide a more accurate assessment of actual mechanical cardiac contractility.

In our study, there were several cases where cardiac activity was demonstrated on PoCUS but not on ECG (5% of asystole cases). Gaspari et al. have previously reported that the detection of cardiac activity on PoCUS is associated with improved clinical outcomes [4]. Their results clearly show that the probability of survival increases in the presence of electrical activity on ECG (PEA) and mechanical activity on PoCUS. As such, it is reasonable to conclude that combining ECG and PoCUS findings may be useful when trying to determine if resuscitation should continue during cardiac arrest. Because patients with asystole and PEA have different outcomes, and ECG alone cannot reliably separate the two in terms of measurable cardiac activity, the addition of PoCUS to ECG may enable us to develop better treatment algorithms for these groups of patients, as it would allow ACLS to further classify “non-shockable” rhythms as either PEA or asystole with their own respective resuscitation guidelines. Caution is required to ensure that the addition of PoCUS would not extend delays in CPR that are required to read both ECG and PoCUS findings [5]. We plan to further explore the combined use of ECG and PoCUS in cardiac arrest.

Limitations

Without video recordings of the ultrasound findings, our ability to closely correlate the timeline for clinical, ECG, and PoCUS findings was limited. Cardiac activity may have changed between these investigations, skewing our interpretation of ECG reliability.

## Conclusions

Our results confirm that although the odds of visualizing cardiac activity on PoCUS are greater for patients with PEA compared to asystole on the initial ECG rhythm and that, as such, ECG rhythm alone is not an accurate predictor of mechanical cardiac activity. Although most patients with asystole on ECG demonstrated no cardiac activity on PoCUS, a small number did have activity. In turn, electrical activity does not accurately predict mechanical cardiac activity. This data supports the use of PoCUS during cardiac arrest, in addition to ECG, to identify patients with ongoing mechanical cardiac activity and to help determine appropriate treatment within the non-shockable group of patients in the ACLS protocol.
